# A Single-Center Audit of Symptomatic Breast Cancer Patient Referrals During COVID-19 Pandemic Restrictions

**DOI:** 10.7759/cureus.26038

**Published:** 2022-06-17

**Authors:** Olukayode M Tokode, Sarah Rastall

**Affiliations:** 1 Oncoplastic Breast Surgery, Princess Royal University Hospital, Telford, GBR; 2 Clinical Anatomy, University of Hull, Hull, GBR

**Keywords:** district hospital, 2-week-wait referral, breast clinic referral, general practitioner, covid-19 pandemic

## Abstract

Background

Recommendations to balance cancer care with patient and hospital staff safety have been issued to hospitals during the coronavirus disease (COVID-19) pandemic. Concerns have been raised that service restrictions could jeopardize effective cancer management. Thus, this study aimed to conduct an audit to verify this proposition.

Methods

We conducted an audit comparing two-week wait (2ww) breast cancer referrals in our center between May and July 2019 and 2020. The primary endpoints were changes in the overall referral rates, differences in the waiting time, and breast cancer diagnosis rates between the two cohorts. Group differences were evaluated using the chi-square test (χ^2^). A p-value of <0.05 at 95% CI was considered significant.

Results

The 2ww referrals decreased by 442 (28.3%) in 2020 (2019 N=1564 vs. 2020, N=1122). Referrals in 2020 were associated with a higher rate of two-week specialist consultation than referrals in 2019 (p<0.05). The 2020 patient cohort was associated with a higher rate of breast cancer diagnosis than the 2019 cohort (6.9% vs. 4.9%, p<0.05). Of the 521 patients who had telephone consultations, 29.2% were discharged, and 367 (70.4%) had post-telephone one-stop clinic visits, of which 9.0% had breast cancer.

Conclusions

The audit provided evidence of effective breast cancer services during the COVID-19 pandemic restrictions. The study results could inform patients and the general public at large that the waiting time and breast cancer diagnosis are not compromised during COVID-19 pandemic management. The high rates of post-telephone one-stop clinic visits and cancer diagnosis may indicate weakness in triage and difficulties in diagnosing nonspecific presentation of cancer over the telephone.

## Introduction

The coronavirus disease (COVID-19) pandemic presents novel challenges to the regular operation of healthcare delivery services worldwide [1-3]. Hospital services were reorganized to balance disease risk and viral exposure to patients and hospital staff. The Association of Breast Surgery of the Royal College of Surgeons of England issued referral guidelines for general practitioners (GPs) [3]. The National Health Service, England, and surgical colleges have recommended modifications to the standard cancer referral protocol [3-5]. Furthermore, national advice and specialist associations’ recommendations have been adapted according to local needs and circumstances to ensure patient and community safety and efficient use of hospital resources [6-7].

The configuration of services to manage COVID-19 has been viewed as skeptical. Concerns have been expressed regarding service restrictions owing to the management of COVID-19, which may lead to delays in cancer diagnosis in general and worsen survival [8-10]. A survey of over 1,000 GPs across the United Kingdom (UK) by Cancer Research UK revealed that a quarter of the respondents asserted that cancer referrals were inappropriately rejected during the COVID-19 outbreak [11]. A previous study [12] reported a 28% reduction in the two-week wait (2ww) breast referral rate in the first six months of 2020 compared with the equivalent period of 2019. However, this study included data from the first three months of 2020, when there were no COVID-19 restrictions [13], and a nationwide dataset that obscured the occurrence in each specialist breast service center. The data used in this study, which was conducted in a district general hospital, were obtained during the COVID-19 lockdown.

We used a prospective breast database to audit the management of 2ww breast referrals during the COVID-19 pandemic. The 2ww is a UK cancer initiative that ensures that all suspected cancer cases are seen by the specialists within two weeks of referrals by the general practitioners (GPs). We compared the overall 2ww breast referrals, waiting times to clinics, and rate of breast cancer suspicion and diagnosis between May-July 2019 and May-July 2020. The relationship between patient demographics and cancer diagnosis was investigated. Additionally, we examined the rate of telephone consultations, the difference in waiting time between telephoned and face-to-face patients, the rate of breast cancer diagnosis among telephoned patients, and the clinic follow-up rate after a telephone consultation.

## Materials and methods

Setting

Specialist breast clinics in our trust have been running for more than two decades. GPs refer patients to breast clinics with a designed pro forma, with or without free text, and approximately 300 patients are referred to our breast clinic every month.

The patients were seen in one-stop breast clinics following the GP’s referrals. All investigations, including ultrasonography, mammography, and core or punch biopsies, were performed at the first clinic visit. Within two weeks of the clinic visit, the biopsy results are discussed at our weekly multidisciplinary team meeting, and the patients are seen at the clinic or telephoned and discharged if all issues are resolved. All symptomatic breast cancer referrals are seen with 2ww priority in our center since an increasing number of breast cancer cases are diagnosed among routine breast cancer referrals [14].

Response to the COVID-19 pandemic

The consultants triaged referrals to the one-stop clinic for suspicious cases or telephone clinics for low-risk cases. Telephoned patients were advised to self-manage and were discharged from the breast service if their issues were resolved or offered a face-to-face follow-up appointment for physical review at the one-stop clinic if further information was required. Some patients were sent for investigations at telephone clinics.

Breast registrars performed telephone consultations, which did not follow a uniform model, and no training in telephone consultation was provided. During the lockdown, surgeries for cancer and postoperative complications were performed, while cosmetic and reconstructive procedures were suspended. The selected patients were managed with hormone therapy as a temporary measure.

Participants

Eligible patients were new symptomatic referrals identified on the prospective breast cancer database during the following study periods: May 1, 2020, to July 31, 2020, and May 1, 2019, to July 31, 2019. The breast cancer database’s information included GP suspicion of cancer, patients’ age and sex, date of referral receipt, date of specialist consultation, consultation mode (face-to-face or telephone), clinical outcome, and diagnosis. The patients’ medical records, including corresponding letters and the results of the investigations, were maintained on the trust website. Patients with new symptoms were included in the analysis. In contrast, rereferrals, dermatological lesions, and referrals from other specialties were excluded.

Data collection

Information collection from the breast cancer database and patient medical records was performed using a data-extraction sheet. The extracted information included patient demographics (age and sex), waiting time to clinic, reason for referral, consultation mode, telephone consultation outcome, month and year of referral, and diagnosis.

Endpoints

Primary endpoints: The primary endpoints include a change in the overall referral rate, difference in the waiting time to clinic, and rate of breast cancer diagnosis between the two cohorts of patients.

Secondary endpoints: The secondary endpoints include the percentage of referrals who underwent telephone consultation in 2020, the difference in the waiting time to the one-stop clinic between telephoned patients and patients who underwent face-to-face consultation, the proportion of patients who underwent clinical follow-up after the telephone consultation, and prevalence of breast cancer diagnosis among the telephoned patients.

Defining the variables

Waiting time to the clinic (days): This was defined as the time from when a referral letter was received to the time of consultation with a breast specialist (face-to-face or telephone). Information was retrieved from the breast cancer database. The length of waiting time was dichotomized to 1 to 14 days (≤ 2 weeks) and 15 days and above (> 2 weeks).

GP diagnosis: This refers to the suspected diagnosis by the GP, described as suspected or unsuspected breast cancer. Information was retrieved from the GP referral letter.

Specialist diagnosis: A benign diagnosis was characterized by normal or hormonal breast changes and benign breast lesions on imaging or pathology. Breast cancer was defined as an invasive carcinoma of the breast, established on imaging and histology.

Consultation mode: This refers to either face-to-face or telephone consultations.

Telephone consultation outcome: Telephone consultation outcome refers to whether the patient was discharged to the GP, scheduled for a different face-to-face clinic after a telephone consultation, or referred for further investigation.

A follow-up one-stop breast clinic: This refers to the face-to-face follow-up consultation offered to patients after the initial telephone consultation.

Statistical analysis

Descriptive statistics are used to describe patient and referral characteristics, and categorical data are reported as percentages. We used the chi-square test (χ2) to evaluate the differences between categorical variables. Differences between variables were considered significant at a p-value lower than 0.05 (two-tailed) at the 95% confidence interval (CI). Statistical analysis was performed using the Statistical Software for Social Sciences (SPSS) version 26 (IBM Inc., Armonk, New York).

Ethics and confidentiality

This audit study was approved by the Trust Audit Department, and informed consent was not required, as anonymized data were used.

## Results

The results of the 2ww referrals are first presented, followed by the results of the telephone consultations.

Characteristics of two-week wait referrals

Participant demographics: Of the 2,686 referrals during the study period (2019-2020), 2,516 (93.7%) were women, and 170 (6.3%) were men. There was no sex difference in referral rates between the 2020 and 2019 cohorts (95% CI: 0.9-1.0, p=0.75). We noted a slight increase in the number of referrals in patients under 30 years of age but a decrease or no increase in the older groups (Table 1).

**Table 1:** Characteristics of two-week wait referrals before (2019) and during (2020) the COVID-19 pandemic

Variable		Pre- and COVID-19 years		Difference (%)
		2019 N=1564 (%)	2020 N=1122 (%)	
Age group (years)	≤ 30	235 (15.0)	179 (16.0)	+ 1.0
	31 - 50	670 (42.8)	480 (42.8)	0.0
	51 - 70	443 (28.4)	308 (27.4)	- 1.0
	≥ 71	216 (13.8)	155 (13.8)	0.0
Waiting time	≤ 2 weeks	1129 (72.2)	1107 (98.7)	+ 25.6
Breast cancer diagnosis	Cancer	76 (4.9)	77 (6.9)	+ 2.0

Primary endpoints

Change in the overall referral rate: There were 1,564 (58.2%) referrals in 2019 and 1,122 (41.8%) referrals in 2020. The number of referrals was reduced by 442 (28.3%) in 2020.

Waiting time in 2019 versus 2020: Patients referred in 2020 achieved higher consultation rates within two weeks than those referred in 2019 (98.7% vs. 72.2% (χ2 = 343.74, df 1, p < 0.05).

Breast cancer diagnosis: Of the 153 patients diagnosed with cancer, 76 (4.9%) were diagnosed in 2019, and 77 (6.9%) were diagnosed in 2020. There was a higher rate of breast cancer in the 2020 cohort than in the 2019 cohort (6.9% vs. 4.9%, χ2=4.83, df=1, p<0.05). No case of cancer was diagnosed in patients aged ≤30 years.

Secondary endpoints

Telephone consultation rate: The telephone consultation was initiated during the COVID-19, coronavirus infection. Of the 1,122 referrals in 2020, 521 (46.4%) were triaged for telephone consultation and 601 (53.6%) for one-stop clinics (Table 2). 

**Table 2:** The two-week wait for breast cancer referrals triaged to telephone and face-to-face consultations during the COVID-19 pandemic in 2020

Variable		Telephone N (%)	Face-to-face N (%)	Difference
Referrals		521 (46.4)	601 (53.6)	+ 7.2%
Waiting time	≤ 2 weeks	515 (98.8)	592 (98.5)	- 0.3
Age (years)	≤ 30	79 (15.2)	100 (16.6)	+ 1.4%
	31 - 50	218 (41.8)	262 (43.6)	+ 1.8%
	51 - 70	142 (27.3)	166 (27.6)	+ 0.3%
	≥ 71	82 (15.7)	73 (12.2)	- 3.5%
Breast cancer diagnosis		33 (6.3)	44 (7.3)	+ 1.0

Telephone versus one-stop clinic waiting time: No significant difference was observed in the telephone and one-stop clinic 2ww time (98.8% vs. 98.5% (χ2=0.19, df=1, p<0.05).

Breast cancer diagnosis: No significant difference in the rate of breast cancer diagnosis was observed between the one-stop clinic and telephone consultation patients (7.3% vs. 6.3% (χ2=0.44, df=1, p>0.05). The stage of the breast cancer was not assessed in this study.

Follow-up rate after telephone consultation: Of the patients who underwent telephone consultations, 152 (29.2%) were discharged, 367 (70.4%) underwent clinical follow-up, and two (0.4%) underwent further investigations. Ninety-one percent (334/367) of patients who underwent follow-up did not have breast cancer, and 33 (9.0%) had a cancer diagnosis.

## Discussion

Overall, the 2ww referrals reduced by 28.3% in 2020 compared with those in 2019. Other studies have recorded fewer urgent and symptomatic referrals during the pandemic [15-18]. We note that there is no difference in the referral rates of patients aged younger and older than 50 years, in contrast to the finding of Bansal et al. [15], which showed reduced referral rates in patients aged more than 50 years. The tie in the referral rates between the two age groups in this study might be due to a relative decrease in the referral rates among the older population. The referral rate fall is linked to several factors, including the GPs more stringent referral approach, health facility avoidance by patients prone to nosocomial infection, and government and media campaign against hospital visits. Our study shows an increased 2ww target and cancer diagnosis rate in the pandemic era, similar to previous findings [15, 16]. The increased 2ww target is partly due to the diversion of facilities and clinicians to the one-stop clinic following the suspension of benign and reconstructive procedures and family history clinics.

There is no statistically significant difference in the wait time and cancer diagnosis rate between the telephone and one-stop clinic patients, suggesting that telephone consultation is not inferior to face-to-face consultation. Several studies have documented the effectiveness of telephone consultation within [16] and outside [19, 20] the breast specialty, although the risk of missed cancers because of lack of clinical examination is always a concern. We observed a high post-telephone one-stop clinic follow-up, and the cancer detection rate of 9.0% of those seen face-to-face post-telephone consult in 2020 is equivalent to routine referral rates [21, 22]. The result may be due to weakness of the triage system or nonspecific breast cancer symptoms, for which face-to-face review is necessary to detect subtle signs of cancer [22]. Doctors' training in telephone triage and the use of a standardized model of consultation may improve the effectiveness of telephone consultations and reduce the rate of post-telephone one-stop clinics.

The improvement in breast cancer survival is linked to screening programs and early diagnosis of symptomatic cases via a 2ww pathway [23]. The COVID-19 pandemic restrictions reduced the breast screening program and modified the symptomatic breast cancer referral policy to facilitate specialist diagnosis and treatment [24]. The results of this study reveal some salient findings. While the shortened 2ww time and increased cancer diagnosis in this study may help ease patients and public anxiety, the COVID-19 pandemic and its restrictions are associated with profound short- and long-term consequences for the patients, hospital organizations, and clinicians. It was anticipated that the reduced 2ww referral rate and breast cancer diagnosis would be followed by a surge in the 2ww referrals and advanced breast cancers after the pandemics [18, 25]. It was also predicted that cancer mortality, including breast cancers, will worsen after the pandemic [8, 10]. Some studies have shown a rebound in the 2ww referral [12, 16, 17, 26] and breast cancer diagnosis rates [16, 25] and predicted a shift in breast cancer diagnosis from early to advanced stages following the COVID-19 pandemic [18, 25]. Several specialist breast services have implemented telephone consultations and virtual meetings during the COVID-19 pandemic [27]. These healthcare delivery approaches are likely to be an integral part of health services, especially during the upsurge of COVID-19. The lessons learned during the pandemic and collaboration between clinicians are essential to make the approaches more effective. The battle with the COVID-19 pandemic is far from over. The emergence of severe acute respiratory syndrome coronavirus 2 strains may stress the hospital workforce as the staff capacity reduces due to isolation and illness, and arrangements to limit viral transmission may slow down the workflow and stress the staff [18].

We have used robust referral and diagnostic data from the breast cancer prospective dataset. The audit results accurately reflect the policy of COVID-19 management on breast cancer referrals and diagnoses in our center. However, our study has limitations. The audit represents the experience of a single specialist breast center, and the results may not be generalizable to other centers. This study has not reported the number of missed cancers among the telephoned and discharged patients, as with previous studies [16, 27]. This study has also not reported the presenting complaints and specific diagnosis data as they are not part of the study design. More research is needed about the consequences and management of the COVID-19 pandemic. Quantitative and qualitative studies on the patient and relative and GP assessments of telephone consultations are required. A larger study on the safety of telephone consultation is also needed. The missed cancer rate of the telephone and discharged patients in our center will be a subject of future research. Further studies are needed to investigate the effect of telephone consultation space on patient-doctor communication. The impact of the pandemic on individuals belonging to different ethnic groups, the poor, and the marginalized within the breast service needs further investigation. 

## Conclusions

The COVID-19 pandemic exerted profound health care and socioeconomic effects worldwide. The study used a prospectively collected dataset to demonstrate the results of our approach to the management of 2ww referrals during the COVID-19 restrictions. The audit results suggest a future increase in the referral rate and the incidence of advanced breast cancer. This also indicates that telephone consultation is as effective as the one-stop clinic in terms of waiting time and cancer diagnosis. The effectiveness of the triage system during the pandemic is partly related to the resilience of breast services and the diligence of clinicians. The COVID-19 pandemic will continue to be the subject of research in the future, and extensive learning during the pandemic will help improve our service organization and delivery in the future. It is expected that the telephone consultation approach may witness the development of best practices and ethical guidelines agreed upon by all stakeholders.
